# Design, execution, and interpretation of plant RNA-seq analyses

**DOI:** 10.3389/fpls.2023.1135455

**Published:** 2023-06-30

**Authors:** Racheal N. Upton, Fernando H. Correr, Jared Lile, Gillian L. Reynolds, Kira Falaschi, Jason P. Cook, Jennifer Lachowiec

**Affiliations:** Plant Sciences and Plant Pathology, Montana State University, Bozeman, MT, United States

**Keywords:** differential expression, co-expression networks, allele-specific variation, QTL mapping, experimental design

## Abstract

Genomics has transformed our understanding of the genetic architecture of traits and the genetic variation present in plants. Here, we present a review of how RNA-seq can be performed to tackle research challenges addressed by plant sciences. We discuss the importance of experimental design in RNA-seq, including considerations for sampling and replication, to avoid pitfalls and wasted resources. Approaches for processing RNA-seq data include quality control and counting features, and we describe common approaches and variations. Though differential gene expression analysis is the most common analysis of RNA-seq data, we review multiple methods for assessing gene expression, including detecting allele-specific gene expression and building co-expression networks. With the production of more RNA-seq data, strategies for integrating these data into genetic mapping pipelines is of increased interest. Finally, special considerations for RNA-seq analysis and interpretation in plants are needed, due to the high genome complexity common across plants. By incorporating informed decisions throughout an RNA-seq experiment, we can increase the knowledge gained.

## 1 Introduction

The production and analysis of transcriptomic data has become the norm in plant sciences. In 2022, over 5700 articles were identified from the search terms “plant transcriptomics” on the PubMed database, and over 23% of the total data available in the Sequence Read Archive is RNA-seq for algae and land plants (Julca et al., 2022). With thousands of public datasets already available and the on-going generation of new transcriptomes, deep knowledge of plant physiology, biochemistry, development, evolution, and more can be gained through careful analysis.

Technologies are rapidly improving to study transcriptomes and becoming more cost effective to be deployed across diverse plants species. While microarrays and RNA-seq are the most common approaches to assess transcriptomes, long-read approaches including Iso-Seq (Schaarschmidt et al., 2020) and Nanopore direct RNA sequencing (Liang et al., 2021; Jain et al., 2022) can also be used to examine transcript isoforms and splicing events. Single-cell RNA-seq (scRNA-seq) can be used to analyze the entire mRNA profile of a single cell, allowing for discovery of new cell types, understanding of cell-to-cell variability, and study of rare cell types (Cuperus, 2022). This review focuses on mRNA transcriptomics as it is the most widely used approach for studying the transcriptome of plants.

For RNA-seq findings to be impactful, careful consideration in the design and analysis of the experiments are critical. In this review, we highlight considerations for new RNA-seq experiments emphasizing experimental design and best practices for processing RNA-seq data. Gene expression determined from RNA-seq represents an endophenotype, molecular phenotypes intermediate to genotype and organismal traits (Mackay et al., 2009). Relating endophenotypes to the underlying genetics and more derived phenotypes is a growing area of research (Mackay et al., 2009; Guo et al., 2016). We therefore, lay out various analyses of gene expression and describe approaches that integrate RNA-seq with gene mapping approaches (Guo et al., 2019; Huang et al., 2022; Jiang et al., 2022) with the intent of increasing the information gained.

## 2 Experimental considerations for successful RNA-seq studies in plants

Thoughtful RNA-seq experimental design is critical for the high-quality data needed to answer complex biological questions. Insufficient experimental design for RNA-seq experiments may lead to makeshift analyses to circumvent inappropriate procedures. These issues have been discussed extensively in human medical literature (Leek et al., 2010; Fang and Cui, 2011; Robles et al., 2012; Williams et al., 2014), but less so in plant sciences. In addition to the standard considerations of experimental design, RNA-seq requires attention to tissue sampling strategy and the impacts of sample processing batch effects. Batch effects refer to the technical artifacts that may be present across a set of samples processed simultaneously. Lack of consistency in sample collection or inattention to batch effects can lead to many complications including lack of statistical power, technical or biological artifacts, and lack of conclusive results. Below we detail considerations that can be instrumental to the success of RNA-seq.

### 2.1 Experimental design concerns

#### 2.1.1 Replication

The definition of a single replicate can vary across laboratory and field-based studies. The experimental unit can range from a single cellular component to a single plant to a collection of multiple plants within a plot, making the definition of sample replicates highly dependent on the system of interest. Similarly, experimental units in RNA-seq experiments can vary and may differ depending on the experimental goal (Conesa et al., 2016). In experimental design parlance, a factor refers to a single categorical variable manipulated in a study. If there is an experiment with one factor with *k* levels, then the study has *k* treatments. When there is a second factor with *j* levels applied in combination with factor *k*, there are *k* x *j* treatments, and so on (Hoshmand, 2006). When multiple experimental units receive the same treatment, they are considered replicates, and contrasts can be made between varying factor levels or treatments.

**Figure 1:**
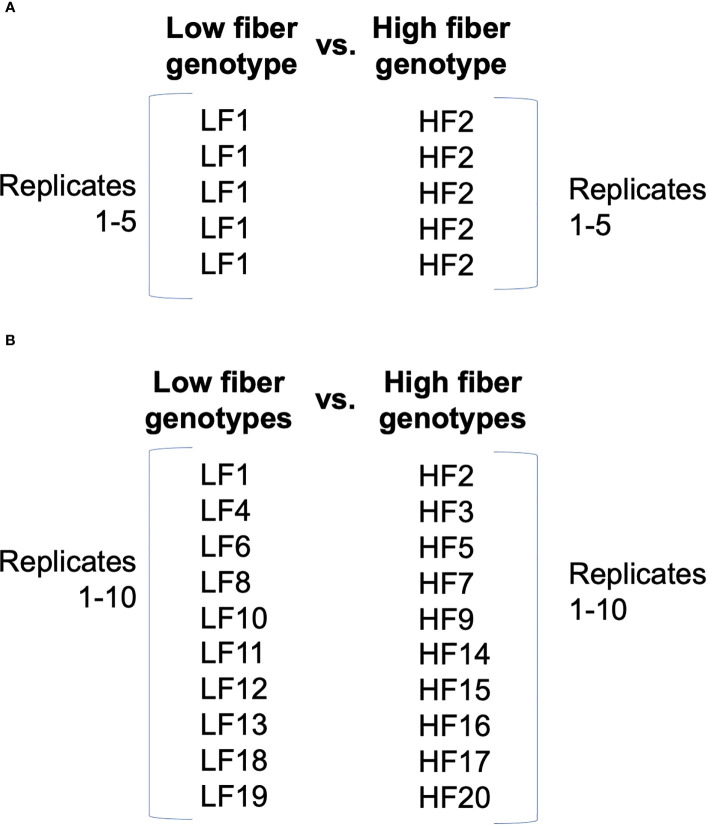
Contrasting definitions of replicates in experimental design. The high-fiber (HF) and low-fiber (LF) classes represent two levels of one factor in the experimental design that are of interest to contrast. Unique genotypes are indicted with numbers. **(A)** Typical design in which the same treatment is applied to multiple experimental units to comprise replicates. **(B)** Alternative design in which different genotypes comprise replicates. Design is from Kasirajan et al. (2018).

With certain experimental goals, like capturing shared patterns of expression, it may be beneficial to more broadly define replicates. For example, if multiple genotypes share the same direction of gene expression change across the levels of another factor, it may be useful to redefine replicates not by a single genotype (**Figure 1A**), but instead across multiple genotypes (**Figure 1B**) (Kasirajan et al., 2018; Correr et al., 2020). Kasirajan et al. (2018) assessed differential expression (DE) between upper and lower sugarcane internodes of two groups of genotypes defined by high or low fiber content, and they used different genotypes within the same fiber content class as replicates. Grouping genotypes with shared functional characteristics allowed the identification of shared candidate regulatory mechanisms.

Once the composition of replicate is determined, the number of replicates to examine is also important for a successful study. In general, replicate number has been found to have a stronger impact on differential expression analysis than sequencing depth (Lamarre et al., 2018). When RNA-seq is used for hypothesis generation, small numbers of samples—usually three—can be sufficient to perform the statistical analysis (Van den Berge et al., 2019). An extensive experiment, exploring the replication needed for RNA-seq, advises 6-12 replicates for each treatment, especially if genes with small changes in expression may be relevant (i.e., transcription factors) (Schurch et al., 2016). Because outlying replicates with poor correlations to other samples can be identified during processing, higher replication can support removing these samples with sufficient replication remaining for downstream analyses (Gierliński et al., 2015).

#### 2.1.2 RNA-seq sensitivity to batch effects

Next-generation sequencing data is highly sensitivity to biological variation and technical artifacts, and RNA-seq is no exception. Minimizing potential sources of unwanted variation during sample collection and preparation enables meaningful interpretation (Zhou et al., 2019). Technical processing of RNA-seq is not only sensitive to the facility and instrument used, but also can be sensitive to the reagent lot, date of sequencing and other processing factors (Molania et al., 2022). Simple experimental design approaches to overcome batch effects include randomization of samples at the library preparation step and across sequencing runs (Conesa et al., 2016). If batch effects are unavoidable, additional experimental design elements can be added, and analysis methods for Removing Unwanted Variation (RUV) can detect and remove batch effects in experiments. RUV approaches use known concentrations of spike-in controls (Risso et al., 2014) to account for batch effects. Additionally, RUV for batch effects can be possible without spike-ins based on presumed housekeeping genes (Risso et al., 2014; Zhang et al., 2020), *in silico* pseudoreplication approaches (Molania et al., 2022), or modeling known sample covariates (Risso et al., 2014).

### 2.2 Sample source considerations

Transcriptomes are highly sensitive, with widespread variability among experimental units exposed to the same treatment detected in even highly controlled environments (Cortijo et al., 2019). Minimizing undesired transcriptional variation can maximize the success of RNA-seq experiments.

#### 2.2.1 Inter-generational impacts on sample sources

It is best practice that all seed needed for an RNA-seq experiment is produced in a common environment the generation prior to the experiment. The growth conditions of the prior generation is an important consideration, as the environments experienced by parental plants can have inter- and even transgenerational effects (Lämke and Bäurle, 2017). A newly formed seed is not a blank slate; the parental environment can affect germ cells and supporting tissues, influence the resulting seed and even the adult plant (Galloway, 2005; Donohue, 2009; Wulff, 2017). For example, parent plants of *Arabidopsis thaliana* grown under higher temperature and radiance produced offspring with higher shoot biomass than parents grown in the lower temperature and radiance condition (Andalo et al., 1999). An RNA-seq experiment designed to compare genotypes could suffer from confounding factors if the environments from which the seed were sourced varied. Another approach to avoid inter-generational effects altogether is to use clones vegetatively propagated from a single individual and apply treatments across clones.

#### 2.2.2 Considerations of composite tissues and bulk RNA-seq

RNA-seq is generally performed on composite samples comprised of multiple tissues or organs, termed bulk RNA-seq. Different tissues and cell types can have varied expression of critical genes that may be masked when measuring pooled transcript levels thus “washing out” the signal of relevant genes in composite samples, which can limit the usefulness of RNA-seq (Johnson et al., 2013). Limitations to biological interpretation with composite samples have been recognized for some time through work focusing on microarray comparisons between cell types and whole tissues. In root cell types identified through reporter gene expression and isolated with fluorescence-activated cell sorting, over 50% of genes with differential expression were repressed in one root cell type and induced in another (Gifford et al., 2008). Pooling of composite cells and tissues may obscure the identification of relevant and causal transcripts that could contribute to improved quality and yield traits but is nearly unavoidable (Schon and Nodine, 2017).

Additional sources of transcriptional variation in composite samples complicate matters further. Varying cell types can simply have different concentrations of total RNA (Baker et al., 1990; Vennapusa et al., 2020; Walsh et al., 2020). For example, within seeds, embryos have high transcriptional activity while the endosperm are less active (Giacomello et al., 2017; Palovaara et al., 2017). Further, RNA is more difficult to extract from some plant tissues and species, such as those rich in polysaccharides or those containing high concentrations of secondary metabolites (Salzman et al., 1999; Gao et al., 2001). Therefore, composite samples with starchy tissues may have transcripts that poorly represent these components due to extraction difficulties. The sources of variation in composite samples can limit the true utility of RNA-seq. Regardless of the challenges of composite tissues studied with bulk RNA-seq, pinpointing the choice of tissue to sample is best informed by the scientific question of interest and hypothesized outcomes supported by independent biological data.

#### 2.2.3 Minimizing diurnal and circadian effects

Throughout the course of a day, temperature, sunlight, and water potential all change, impacting plants (Hotta, 2021). Circadian variation is of particular importance when considering photosynthetic tissues. In plants, changes in the transcriptome due to circadian rhythm can cause a 25% fluctuation in differentially expressed genes (Hayes et al., 2010; Hudson, 2010), and mRNA decay rates in *Arabidopsis thaliana* vary widely from just under four minutes to over 24 hours with median half-lives of 2-4 hours (Narsai et al., 2007; Sorenson et al., 2018). Therefore, the timing of RNA-seq studies must be highly controlled during sample collection. In studies where there are multiple treatments and circadian rhythm is not the focus, samples need to be collected at the same time to avoid confounding variance due to time of day. Similarly, for temporal studies across multiple days, samples need to be taken at the same time of day. For some species, a database of circadian controlled gene expression is available for post-hoc correction (Li et al., 2016), but a conscientious design is a superior approach.

## 3 From reads to genetic features

After thoughtful RNA-seq experimental design, sample collection, and generation of RNA-seq reads, it can be overwhelming to approach the myriad of different tools available for analysis. In this section we aim to provide a general overview of the different approaches for quantifying the expression of genetic features (genes or transcript isoforms) from raw sequencing reads. The software available are categorized by purpose—quality control, alignment, or quantification—and the genomic information available for the organism. The software described below is commonly used for RNA-seq pipelines; however, this is not a complete list of all software as the tools available are numerous and new software is being developed rapidly (Van den Berge et al., 2019).

### 3.1 Preprocessing and quality control

Preprocessing comprises the first step for any sequencing analysis. This stage involves the removal of technical artifacts such as adaptors, PhiX sequence, rRNA sequences, assessing sequence quality and if necessary, quality trimming. Commonly, sequencing centers perform the preprocessing steps of the RNA-seq pipeline, but it is best practice to perform a data quality check in-house. A multitude of tools are available for the preprocessing steps. Quality of raw sequences can be checked via tools such as FastQC (Andrews, 2010) and MultiQC (Ewels et al., 2016) or fastp (Chen S. et al., 2018). Preprocessing tools such as such as Cutadapt (Martin, 2011), Trimmomatic (Bolger et al., 2014), BBTools (Bushnell, 2022), and fastp (Chen et al., 2018) can be used for contamination removal or quality trimming. rRNA contamination can be identified and removed with tools like BBTools and SortMeRNA (Kopylova et al., 2012).

Preprocessing steps should be implemented with caution, as they impact downstream analyses. A more stringent read trimming shortens reads and thus influences mapping and the estimates of expression levels for genes and their isoforms, ultimately impacting the differential expression analysis (Williams et al., 2016). For *de novo* transcriptome analysis, a stringent trimming strategy has also been observed to produce incomplete transcriptome reconstruction (MacManes, 2014; Mbandi et al., 2014). To ensure the greatest amount of information is retained for analysis, employing less stringent read-trimming, or no read-trimming at all is suggested (Del Fabbro et al., 2013; MacManes, 2014; Mbandi et al., 2014; Williams et al., 2016).

### 3.2 Alignment and reconstruction: reference-based and *de novo* approaches

The next step in the RNA-seq analysis pipeline is to align reads using a reference genome or *de novo* methods. Similar to preprocessing steps, there are many bioinformatic tools available for aligning reads, and they are categorized by whether they require a reference genome or a *de novo* transcriptome. In this section we provide a brief overview of the different methods available for both reference and *de novo*-based approaches, with a focus on the types of biological questions each method can address and the limitations of each approach.

Alignment-based reconstruction occurs when a reference genome is available. The approach is very similar to aligning genomic reads, but splicing events need to be considered for RNA-seq data (Mehmood et al., 2020). Some of the software available to perform the reference-based alignment includes HISAT2 (Kim et al., 2019) and STAR (Dobin et al., 2013). Reference-based alignment is split into two parts: reference genome indexing and alignment of reads to the indexed reference genome. Additionally, some alignment tools can incorporate the discovery of novel exon-exon junctions like HISAT2 (Kim et al., 2019) and RsubRead (Liao et al., 2019).

One drawback to reference-based alignments is reference-bias: if there are sequences present in the RNA-seq data that are not present in the reference data, the data will not align and will be lost for downstream analyses unless alternative mining is performed (Yang et al., 2022). A reference-based transcript reconstruction can be performed post-alignment by software like Cufflinks (Trapnell et al., 2010), StringTie2 (Kovaka et al., 2019) and Bookend (Schon et al., 2022). Alternatively, new approaches that align reads to pangenomes are available. Pangenomes store population-level genetic variation into a graph-based structure rather than a single linear genome (Eizenga et al., 2020), allowing for improved read alignment, including for haplotype-aware RNA-seq read alignment (Sibbesen et al., 2023).

When a reference genome or pangenome is not available for your species of interest, researchers can choose to perform a *de novo* assembly transcriptome. *De novo* transcriptome assembly can be performed by tools as Trinity (Grabherr et al., 2011) and TransLiG (Liu et al., 2019). The same tools for a reference genome-based approach are used to align RNA-Seq to a *de novo* transcriptome with minor modifications – e.g., not using the splice-aware function of the aligners.

The next step in the bioinformatics pipeline is counting reads mapped. For reference-based approaches tools such as HT-Seq (Anders et al., 2015) or featureCounts can be used (Liao et al., 2014). Also, transcript quantification using a reference transcriptome can be performed using *alignment-free* methods like Kallisto (Bray et al., 2016) and Salmon (Patro et al., 2017). It is worthwhile mentioning that RNA-Seq strandedness impacts quantification, identification of isoforms and *de novo* transcriptome assemblies. The concept refers to the strategy employed in library construction, wherein stranded library preparations maintain the transcript directionality. Hence, researchers should be aware of the kit utilized for library construction when performed the aforementioned steps of RNA-seq analysis. When the information of strandedness and direction of strandedness is not known, tools like how_are_we_stranded_here (Signal and Kahlke, 2022) can help determine the strandedness of paired-end libraries. Finally, tools like tximport (Soneson et al., 2015) and tximeta (Love et al., 2020) can be used to summarize the quantification of transcript abundances into an expression matrix. For a detailed review about alignment and quantification, we refer the reader to Van den Berge et al. (2019).

## 4 DEGs and beyond: RNA-seq analysis types

Once RNA-seq transcript abundance has been acquired, a multitude of analyses are available to examine a transcriptome. Most often, treatments are contrasted for the identification of differentially expressed genes (DEGs). We also detail additional analyses that can be performed to further understand transcriptional profiles.

### 4.1 Differential expression

Measuring plant phenotypic plasticity in extreme environments can aid in knowledge to develop plants for future abiotic and biotic environmental conditions arriving with climate change (Gage et al., 2017; Monforte, 2020). Using various molecular mechanisms, plants respond to changing environmental conditions by altering their physiology and development (Lachowiec et al., 2015). Molecular plant plasticity enables adaptation to climatic shifts and predicts an individual’s survival success (Nicotra and Davidson, 2010; Nicotra et al., 2010; Fox et al., 2019; Anderson and Song, 2020; Pazzaglia et al., 2021), warranting further investigation (Brooker et al., 2022).

Increasingly studies use RNA-seq to understand molecular plant plasticity (Kumar et al., 2022; Sreeratree et al., 2022). The primary approach analyzes differential expression (DE), which evaluates the transcriptional abundance across conditions through simultaneous statistical testing for significant changes in expression levels in all detected genes, transcripts, or different usage of transcripts/exons (Soneson et al., 2015; Van den Berge et al., 2019). Software like edgeR (Robinson et al., 2010), DESeq2 (Love et al., 2014), and limma (Ritchie et al., 2015; Stark et al., 2019) all provide robust DE analyses. For reviews of the main aspects in the differential expression analysis we refer the reader to Costa-Silva et al. (2021); Stark et al. (2019) and Van den Berge et al. (2019).

Differential expression analysis incorporates a matrix of features’ expression levels and knowledge about the experimental design. Tests for identifying differentially expressed genes (DEGs) rely on contrasting conditions, such as different tissues, genotypes, and conditions. Exploring the up- or downregulation of genes under a stress condition relative to a control condition indicates how a plant combats a stressor and how the stressor harms the plant. For example, in the sorghum lateral root apex, low levels of phosphorus caused major expression changes in the lateral root apices, which correlated with enhanced lateral root growth. Specifically, the low-phosphorus-induced genes encoded proteins with functions in nutrient responses and contribution to phosphorus metabolism (Gladman et al., 2022). Contrasting genotypes with different performances under stress enables identifying potential mechanisms of stress resistance (Yue et al., 2016; Zhao et al., 2021).

### 4.2 Allele-specific expression

Allele-specific expression (ASE) describes the phenomenon where alleles within a particular genetic feature (e.g. gene, transcript) have significant differences in their expression levels (Castel et al., 2015). The expression of alleles can be assessed by comparing the expression of genes of a certain genotype with its parents or by identifying polymorphisms and quantifying the expression of each allele. ASE is especially informative in understanding hybrid crops where different parental genotypes are combined (Bell et al., 2013; Shao et al., 2019). ASE analysis also reveals the processes of genetic imprinting, tissue- and stress-specific alleles, as well as the evolution of species.

In wheat seeds, expression of homeologs and alleles is differentially controlled and consideration of each copy of a gene is relevant. In the endosperm, genes exhibited subgenome dominance in particular functions (Pfeifer et al., 2014). Further, imprinted genes were identified more frequently in developing endosperm relative to other tissues, and imprinted gene expression patterns were conserved through wheat evolution (Yang et al., 2018). This imbalanced expression of maternal and paternal alleles and subgenome dominance supports proper seed development.

ASE analysis uncovered genes that were targets of selection during domestication with implications for plant sciences. Lemmon et al. (2014) identified that maize and teosinte diverged in gene expression especially due to *cis* regulation, where the expression of maize alleles is favored in F1 hybrids between the species. Genes with *cis* and *cis* plus *trans* divergent regulation were enriched among putative targets of selection. In rice, a F1 hybrid of genotypes representing two major subpopulations exhibit enrichment of genes ASE in genomic regions of signatures for domestication or artificial selection (Shao et al., 2019). Moreover, the limited ASE in sugarcane internodes predominates as genotype-specific phenomenon, favoring high dosage alleles and purging the expression of potentially deleterious alleles (Margarido et al., 2022). Detecting ASE and regulatory mechanisms of ASE can inform our understanding of plant evolutionary history.

### 4.3 Alternative splicing

Alternative splicing of precursor mRNAs leads to diversification of the functions of a single gene. Transcriptional or isoform switching refers to a shift in the presence or dominance of transcripts in different samples, including across cell types, development, genotypes, and environments. Within plants, the most common form of alternative splicing is intron retention (Chamala et al., 2015), in contrast to animals where exon skipping is most detected (Kim et al., 2007). When the chosen alignment and counting approaches (see Section 3.2) enable distinguishing transcripts, examination of transcript switching is possible by utilizing the differential expression of individual transcripts or using accessible tools for assessing transcriptional switching (Qiu et al., 2021).

Genome-wide surveys of alternative splicing demonstrate the potential impact of alternative splicing events, with tens of thousands of alternative splice forms detected that are evolutionarily dynamic across angiosperms (Chamala et al., 2015). Across a population of over 350 inbred maize lines, variation in alternative splicing was detected, highlighting that connecting genotype to phenotype can be better informed by considering the expression of particular splice forms (Chen Q. et al., 2018).

### 4.4 Co-expression networks

Co-expression summarizes large-scale transcriptomics to infergene regulatory networks by identifying modules of genes with similar expression patterns across multiple samples. Co-expression analyses can suggest target genes of interest and corroborate GWAS results and expand potential genetic markers from those findings as well. Putative functions then can be assigned to non-annotated genes if the majority of the genes in a module share similar biological functions, the guilt-by-association principle (Serin et al., 2016; Rao and Dixon, 2019). Co-expression network analyses split into two main approaches: (i) non-targeted—a network based on the topological structure using the relationship of all pairs of genes or (ii) targeted—the use of bait genes as prior information to define network connections. Varied inputs are used to build networks, including replicates of multiple treatments, averaging the expression of replicates grown for a treatment, or defining networks separately to single levels of the experimental factor (Cortijo et al., 2020).

A fundamental step for inferring the co-expression of genes from large-scale transcriptomic data is the use of similarity measures, including correlation and mutual information methods (Ma and Wang, 2012; Huang et al., 2017). Correlation methods selection is based on data type, and common coefficients include Pearson’s, Spearman’s and Gini’s (Huang et al., 2017). Gini’s correlation, for example, is advantageous for nonnormally distributed RNA-Seq data, robust against outliers and small sample sizes (Ma and Wang, 2012). A common pipeline to construct a co-expression network involves the calculation of a similarity matrix that is then filtered based on a threshold to select gene pairs; then an adjacency matrix can be calculated and subsequently a clustering algorithm is used to group genes into modules (Serin et al., 2016).

Studies use co-expression networks to find hubs—genes with high network connectivity—and understand their role in biological pathways. For example, sugarcane hub genes changed across four stages of development in the networks of 10-month-old compared to 6-month-old apical culms (Hosaka et al., 2021), revealing candidates related to cell wall and stress and three transcription factors (TFs) potentially acting as regulators of those processes. Co-expression networks may also uncover functions for uncharacterized proteins. De Vega and colleagues (De Vega et al., 2021) inferred TF targets in Miscanthus hybrids that were enriched with carbohydrate metabolism, secondary metabolism, and the generation of precursor metabolites. They also found two TF that linked a core and a loop subnetwork, the last composed mostly by TFs linked to uncharacterized genes. Thus, regulatory co-expression networks are useful tools to identify targets of TFs, which can be important targets for biotechnology and propose functions for poorly understood proteins (Simons et al., 2006; Fröschel et al., 2019).

Time-series expression data impose a challenge for understanding the dynamics of the coregulation of genes. The complex relationships arising due to time can be detected by gene co-expression measures that account for local dependence structures in the expression patterns (Wang et al., 2014). The dynamic network biomarker approach (Chen et al., 2012) aims to find a subnetwork of strongly correlated genes just before a critical transition – identified as a tipping point. With this approach researchers found genes at the tipping point for response to stress or ripening of fruits (Wang and Zhang, 2021; Wang et al., 2022).

Co-expression networks can also provide additional data for determining genes involved in regulatory networks. Co-expression combined with ChIP-seq can also identify the targets of a TF. Cortijo and colleagues (Cortijo et al., 2020) identified novel regulatory targets in the *Arabidopsis thaliana* circadian clock by combining modules with genes co-expressed with known TFs and ChIP-seq data. Targets of PSEUDO-RESPONSE REGULATOR 5—a core component of the circadian clock—were found in a module showing enrichment for photosynthesis. For a complete review of co-expression networks in plant biology, the reader is referred to (Rao and Dixon, 2019) and (Serin et al., 2016).

### 4.5 Pathway enrichment

Enrichment analysis tests if any functional group is over- or underrepresented by a list of genes of interest – e.g., DEGs, co-expressed genes or ASE genes. Enrichment analysis of genes identified by differential expression or modules in co-expression networks are useful to understand if genes in the module are related to similar functions. It can suggest that non-annotated genes likely participate in the same biological pathways as known genes, which can lead to the identity of causal genes (Serin et al., 2016). Functional pathways are represented by ontologies in different frameworks like Gene Ontology (GO) categories (Ashburner et al., 2000), Kyoto Encyclopedia of Genes and Genomes (KEGG) orthology (KO) (Kanehisa et al., 2017), and MapMan4 bin categories (Schwacke et al., 2019), which all capture varied functions of genes.

For plants with reference genomes, functional annotation is provided by databases along with the nucleotide sequence and the structural annotation. For *de novo* assemblies this procedure requires comparisons with nucleotide or protein databases, retrieving the functional categories from the hits and associating them to genes/transcripts of the reference. While functional annotation of a reference – genome or transcriptome – in terms of GOs, Enzyme Codes (ECs) or KOs can be performed by tools like OmicsBox (BioBam Bioinformatics, 2019) or Trinotate (Bryant et al., 2017), Mercator4 provides the specific annotation for MapMan (Schwacke et al., 2019). The KO/EC annotation can be linked to molecular networks by KEGG Mapper (Kanehisa and Sato, 2020) to visual representation of the pathways. MapMan4 also has its own visualization of functional pathways where besides the category mapping file, fold change or expression values can be provided as input.

Finally, tests for enrichment of categories—over or underrepresentation—frequently make use of Fisher’s Exact test, Hypergeometrical test, permutation, or variation of those methods. There are many tools for functional enrichment analysis: goseq (Young et al., 2010), GSEA (Subramanian et al., 2005), OmicsBox (BioBam Bioinformatics, 2019), topGO (Alexa et al., 2006), clusterProfiler (Yu et al., 2012), and agriGO (Du et al., 2010).

### 4.6 Viral discovery

Viruses are the most abundant biological entities on earth and are strongly associated with all organisms (Rosani and Gerdol, 2017; Mushegian, 2020). It is very common for viruses to contaminate tissue when preparing samples for sequencing. One meta-analysis found that over half of the 700 sequencing libraries examined had viral contamination (Asplund et al., 2019). RNA-seq is susceptible to viral contamination. However, most viral contamination is filtered out during bioinformatic processing.

Viral contamination in RNA-seq studies can provide informative results as well. Kamitani et al. (2016) looked at both plant-virus and virus-virus interactions in natural environments using RNA-seq. Other studies have used RNA-seq to screen for viral pathogens (Selitsky et al., 2020) and novel viral genome discovery (Rosani and Gerdol, 2017). However, some virus types require alternative rRNA depletion methods to be detected, such as non-polyadenylated genomes (Nagano et al., 2015). To capture the full range of viral genome varieties Nagano et al. (2015) optimized rRNA depletion methods for mRNA focused RNA-seq to detect varying types of viral signatures from natural environments. Viral contamination in transcriptomic data may provide insight to unknown biotic stressors or uncover novel defense mechanisms related to a previously unknown viral pathogen. Accessible software for identifying the species of origin allows for examining unmapped sequencing reads and examining expression patterns for additional species (Chen et al., 2020).

## 5 RNA-seq: from candidate to causal gene

A major goal in plant genetics is to identify alleles or genes that control a trait by associating genotype with phenotype, using both reductionist and holistic approaches. In quantitative trait locus (QTL) mapping, genetic loci that determine a trait are examined one-by-one, typically focusing on loci of major effect to find genotypes with a desired allele. In contrast, genomic prediction collectively considers the impact of the entire genotype on a focal trait, especially impactful for traits controlled by many loci of small effect (Meuwissen et al., 2001).

Within a plant, the genotype is filtered through many levels of endo- or molecular phenotypes to create phenotypes of interest (Mackay et al., 2009). Integrating molecular phenotypes, such as gene expression-, protein- or metabolite-level information with genotypes can improve understanding the association between genotype and phenotype in QTL mapping.

RNA-seq supports fine mapping QTL, both by providing additional evidence for the causal gene via differential expression data and by allowing the discovery of new variants in the QTL region, which can be used for marker development (Liu et al., 2016; Habib et al., 2018; Jaganathan et al., 2020). A suitable situation for using RNA-seq in a fine mapping project is one designed in such a way that a differentially expressed gene within the target QTL is the gene of interest. One technique to maximize this possibility is by generating a pair of near-isogenic lines (NILs) which are genetically identical except for the QTL genotype, and by extension, gene expression and phenotype (Szalma et al., 2007). Using NIL pairs will minimize background genetic noise, so that differentially expressed genes between the pair are likely associated with the trait of interest (Keurentjes et al., 2007). Candidate genes are those occurring in the QTL region that are differentially expressed between the NIL pair. Subsequent analysis of each candidate gene can include using molecular techniques such as gene silencing to observe phenotypic changes as well as enrichment analysis to infer the genes’ function. In addition to expression analysis, RNA-seq data can be used to identify variants in the QTL region which can be used to develop new markers to further increase the resolution of the region (Paritosh et al., 2013). Taken together, the ability of RNA-seq to both provide evidence for the causal gene through expression data and to allow for the identification of new variants in the QTL region for marker development, means that it can be a powerful tool for fine mapping applications.

Similarly, combining co-expression networks with genome-wide association studies (GWAS) has prioritized candidate genes (Chan et al., 2011). In contrast to biparental QTL mapping, GWAS relies on diverse accessions that show phenotypic variation in a trait of interest. Schaefer et al. (2018) developed a framework to integrate GWAS for maize grain ionome traits and co-expression networks leading the identification of two important genes expressed in roots (Schaefer et al., 2018). The maize *dwarf9* (*d9*) dominant allele *D9-1* had higher abundance of elements like iron, sulfur, and strontium compared to the wild type. *D9-1* did not influence cadmium accumulation, as expected by the location of the cadmium GWAS QTL that contained *d9*. Rather the dwarf allele –*D8-mpl* – of the paralog *d8* identified through co-expression analysis, recapitulated this effect. Co-expression networks can also support extending understanding of the genetic architecture of traits through the examination of epistasis. A maize GWAS for senescence-controlling genes identified putative epistatic interactions among QTL that were independently supported with co-expression data (Sekhon et al., 2019). Co-expression networks integrated with mapping approaches are proving to be powerful for the identification of important genes and genetic architecture (Rao and Dixon, 2019).

## 6 Genome complexity and RNA-seq in plant sciences

Aspects of plant genome biology complicate the use of RNA-seq data, especially relative to the organisms for which many of the bioinformatics tools were developed, though efforts to produce specific tools for polyploid crops are expanding (Foster et al., 2019). The genomes encoding many plants are polyploid with multiple subgenomes (Meyer et al., 2012). Additionally, plants can harbor massive levels of repetitive sequences (Zimin et al., 2014).

### 6.1 Polyploidy

One feature of plant genomes that can influence the use of RNA-seq is the prevalence of polyploidy (Fu et al., 2016). A study of 203 modern crops identified that 17% have undergone polyploidization (Meyer et al., 2012). Of these, wheat and sugarcane are among the most cultivated crops globally (Weeks, 2017). The pairing of polyploid chromosomes during recombination varies along a spectrum from allopolyploidy to autopolyploidy, along with the presence of aneuploid chromosomes. Within allopolyploids, often formed through hybridization, chromosome copies behave in a diploid fashion and pair and segregate corresponding to species of origin, within subgenomes (Edger et al., 2018; Kuo et al., 2020). In contrast, homologous autopolyploid chromosomes pair at random, even between subgenomes (Spoelhof et al., 2017). The type of polyploidy can also vary along the length of a single chromosome. Polyploidization impacts the bioinformatic pipelines used to assess RNA-seq data, both at the level of processing reads and subsequent analyses. The presence of polyploidy can be problematic for RNA-seq analysis, from both a computational and biological standpoint.

Polyploidy increases computation complexity due to an increase in genome size, relative to a diploid genome and the increased repeat content. The increase in genome size, resulting from the presence of two or more subgenomes, means more memory is required to index and store the reference genome or transcriptome prior to read alignment. As such, analyzing large, polyploid genomes, such as wheat, may be prohibitive to those without expansive compute resources.

The degree of similarity among polyploid subgenomes is a driving force for how RNA-seq analyses may need to proceed differently compared to diploid genomes (Voshall and Moriyama, 2020). In the cases of subgenomes with low levels of divergence at the nucleotide level, little functional divergence may be expected across homeologs, the genes homologous to one another on each subgenome (Wang et al., 2017; Sigel et al., 2019). However, the phenomenon of subgenome dominance, or the tendency for genes to be expressed from a particular subgenomes, is commonly observed across plants (Schnable et al., 2011; Wang X. et al., 2016; Khan et al., 2020). For example, nonbalanced expression was identified in approximately ~30% of wheat homeologs, primarily through suppression of a single homeolog (Ramírez-González et al., 2018). Isolating the expression of homeologous genes may be particularly useful when using RNA-seq to complement QTL mapping studies (Yang et al., 2014). Therefore, distinguishing homeolog expression enables specific studies.

### 6.2 Repetitive sequences

High levels of repeat content are also problematic from a computational viewpoint to interpret RNA-seq data. Repeat content can refer to both biological repeats (e.g., transposable elements, short sequence repeats) or genomic regions shared between subgenomes. Significant portions of widely grown cereals are comprised of repetitive sequences, excluding homeologs and duplicated genes: wheat-85% (Wicker et al., 2018), maize-85% (Schnable et al., 2009), barley-80% (Wicker et al., 2017), and rice-41% (Li et al., 2021). In the cases when these regions are expressed (Cavrak et al., 2014), they create issues computationally as there may be no way to confidently align reads to regions that are present more than once, resulting in a high number of multi-mapping reads. Multi-mapping reads are discarded by default by popular read counting programs. As such, the RNA-seq analysis of polyploid genomes may be hampered by the loss of a significant portion of data without explicit intervention.

Data loss due to repetitive sequences is problematic as repetitive elements can have important biological functions. For example, screening *A. thaliana* T-DNA insertion lines at the locations of transposable elements for seedling morphological responses to stresses uncovered functional roles of for over 90% of those tested (Joly-Lopez et al., 2017). Outside of transposable elements, short tandem repeats contribute to the repetitive content of the genome with functional consequences. The length of the short tandem repeat encoding a polyglutamine span in the protein *EARLY FLOWERING 3* influenced flowering time across *A. thaliana* accessions (Undurraga et al., 2012). Inability to assign reads to these repeats means that functional polymorphisms are missing from analyses. Longer read technologies will improve reference genomes (Jiao et al., 2017; Michael et al., 2018; Kamal et al., 2022) and transcriptomes to enable study (Wang B. et al., 2016) of currently poorly characterized sequences.

## 7 Conclusions

The ever-increasing need to support a growing global population requires increases in plant productivity and access. New crops and varieties are needed to adapt to challenging abiotic and biotic environmental conditions globally. With the growing affordability and accessibility of RNA-seq, this technology can be leveraged to study any plant species of interest. The most impactful RNA-seq studies are carefully designed and control many sources of unwanted variation. During analysis, much research concludes by examining DEGs between conditions; however, we have outlined additional forms of analysis to extend the usefulness of RNA-seq data, even data already publicly available. The intent of this review is to provide plant-focused guidelines, strategies, and examples for supporting new users of RNA-seq and inspiration for new applications by established researchers.

## Author contributions

RNU, FHC, JLi, GLR, KF, JPC, and JLa drafted and finalized themanuscript. All authors contributed to the article and approved the submitted version.
